# Editorial: Complex Scenarios of Drug-Resistant Epilepsies: Diagnostic Challenges and Novel Therapeutic Options

**DOI:** 10.3389/fneur.2022.908163

**Published:** 2022-04-28

**Authors:** Giuseppe Didato, Valentina Chiesa, Emma Losito, Ricardo Amorim Leite, Taylor J. Abel

**Affiliations:** ^1^Epilepsy Unit, Fondazione IRCCS Istituto Neurologico Carlo Besta, Milan, Italy; ^2^Epilepsy Center, San Paolo Hospital, ASST Santi Paolo e Carlo, Milan, Italy; ^3^Department of Clinical Neurophysiology, Assistance Publique - Hôpitaux de Paris, Necker-Enfants Malades Hospital, Paris, France; ^4^Video-EEG Unit, Psychiatry Institute of São Paulo University—USP, São Paulo, Brazil; ^5^Department of Neurological Surgery, University of Pittsburgh, Pittsburgh, PA, United States; ^6^Department of Bioengineering, University of Pittsburgh, Pittsburgh, PA, United States

**Keywords:** drug-resistant epilepsy, epilepsy surgery, non-resective epilepsy surgery, multifocal epilepsy, neurostimulation

Epilepsy affects about 70 million people worldwide and in 60% of these patients the origin of epileptic seizures is due to a localized (focal) alteration of the brain. In about 30% of patients with focal epilepsy, drug treatment is ineffective, a condition defined by the International League Against Epilepsy (ILAE) as drug-resistant epilepsy (DRE) (1).

A significant proportion of patients with medically refractory focal epilepsy can benefit from a surgical treatment, which offers the chance of seizure remission in many cases or decrease in seizure frequency and severity (2, 3). The percentage of patients with epilepsy completely cured after surgery is about 70% for temporal lobe epilepsies (2–4), and can reach up to 90% in the case of some brain malformations and tumors (5, 6).

Unfortunately, in certain situations traditional epilepsy surgery approaches are not possible, which include bilateral or multifocal seizures, involvement of eloquent cortical areas, or with associated surgical comorbidities (7, 8). However, recent alternatives to traditional non-pharmacological treatments, other than resective surgery, are now a therapeutic option for these patients.

Recently, novel pre-surgical diagnostic methods and new surgical approaches have been developed. Further, a precise definition of the epileptogenic network can provide an opportunity for surgery in complex refractory epilepsies. For patients who are not good candidates for resective treatments, the evolution in neuromodulation devices and other non-resective surgical procedures also offer good chances of seizure control and improved quality of life (3, 9).

During pre-surgical evaluation for conditions such as multifocal or bitemporal epilepsy, periventricular nodular heterotopia (PNH), tuberous sclerosis complex (TSC), Rasmussen encephalitis, seizures arising from eloquent areas, the localization of the ictal onset zone encompasses diagnostic challenges that can be overcome by means of advanced neurophysiological and radiological methods. This can allow for the epileptogenic zone (EZ) identification of difficult-to-localize focal epilepsies (10, 11).

Furthermore, such complex forms of intractable focal epilepsies are challenges for resective epilepsy surgery. Therefore, recent enhancements in surgical techniques, such as laser interstitial thermal therapy (LiTT), magnetic resonance-guided focused ultrasound (MRgFUS), radiofrequency thermocoagulation (RF-THC), radiosurgery (cyber-knife, gamma-knife) can enable surgical treatment for these patients, targeting the EZ even when this is difficult to approach using classical surgical procedures. Moreover, these new techniques can minimize surgical risks, making surgery possible for patients who would otherwise not be candidates due to their comorbidities (3, 12–14).

For patients without possibility of surgical access to the primary EZ, neuromodulation therapies have been an option for several years. However, new developments in technologies and the increasing knowledge of the circuits involved in neuromodulation have expanded the accomplishment of these treatments. The main advanced neurostimulation technologies are Responsive Neurostimulation (RNS^®^), Deep Brain Stimulation (DBS) and Vagus Nerve Stimulation (VNS) (15–18).

In this Research Topic of Frontiers in Neurology, we have brought together experts in these new areas of epilepsy research, diagnosis, and treatment. This Research Topic provides a balanced collection of eight original research studies, four case reports, one review, and two perspective articles.

The first article, from Wu et al. (Chicago, USA), provides a systematic overview on the seizure outcome of stereotactic laser amygdalohippocampectomy in 30 consecutive patients with mesial temporal lobe epilepsy (mTLE). They demonstrate that scalp EEG findings (interictal regional slow activity on the side of surgery and/or non-lateralizing or contra-lateralizing seizure onset) strongly predicted seizure recurrence after surgery.

Consales et al. (Genoa, Italy), focus on other important indications for laser ablation. They report their experience with six pediatric cases of hypothalamic hamartomas, one case of TSC, and one case of dysembryoplastic neuroepithelial tumor, treated with magnetic resonance-guided laser interstitial thermal therapy (MR-gLiTT). Taken together, their data show that MR-gLiTT is a highly safe and effective procedure for these epilepsy conditions in children.

Welch et al. (Pittsburgh, USA), provide information on the effectiveness of RNS in a 16-year-old male with drug-resistant primary generalized epilepsy with convulsive and absence seizures. This case report demonstrates that bilateral RNS of the centromedian nuclei brought to a complete resolution of the baseline daily absence seizure activity, and to a significant decrease in convulsive seizures.

Ma et al. (China) contribute to the Research Topic describing the use of a new method, named spatio-temporal unifying tomography (STOUT), applied to magnetoencephalography (MEG) to locate a deep source in a patient with insular epilepsy. With this case study they demonstrate that MEG STOUT method can allow the epileptologist to perform a stereo-electroencephalographic (SEEG)-guided RF-THC operation in the event of deep sources, achieving a satisfactory therapeutic effect.

Dimova and Minkin (Sofia, Bulgaria) enrich this Research Topic reporting on a patient affected by a drug-resistant epilepsy involving limbic structures on the left side and associated to anti-GAD65 autoantibodies positivity. The authors describe how the patient benefited from a combination of immunotherapies and surgical treatment (selective amygdalectomy). This case report sheds light on the possibility to consider epilepsy surgery even in patients with complex etiologies.

Iimura et al. (Japan) add another contribution to this Research Topic illustrating how subtotal hemispherotomy dramatically improved epileptic spasms (ES) of a 3-month-old patient with Aicardi syndrome (AS). The authors provide intraoperative neurophysiological evidence as a possible explanation of the successful procedure. They hypothesized that electrocorticography HFOs and phase-amplitude coupling of HFOs and slow wave bands before and after subtotal hemispherotomy could be the biomarkers of efficacy of this surgical procedure in AS with ES.

In addition to the contribution given by the aforementioned authors with regard to possible surgical treatments in complex epilepsy cases, other authors have provided important inputs on new diagnostic procedures aimed at improving EZ localization in focal epilepsies.

The series of contributions to this Research Topic on implemented diagnostic methods in epilepsy opens with an original study by Bacon et al. (China). These authors compare two different tools for quantitative analysis of fluorodeoxyglucose positron emission tomography (FDG-PET) images: statistical parametric mapping (SPM) and its computational anatomy toolbox (SPM-CAT). They demonstrate that SPM-CAT is more efficient than SPM in localizing EZ for DRE. Therefore, this paper underlines the importance of quantitative analysis of FDG-PET images as an objective complementary tool to the visual assessment for EZ localization.

Müller et al. (Bern, Switzerland) highlight how quantitative EEG analysis can enhance the accuracy of identification of the brain tissue generating epileptiform events. They showed that non-linear intracranial EEG analysis may provide information relevant for surgery planning in temporal lobe epilepsy.

Ganti et al. (India) add to this section of the Research Topic their experience on seizure detection algorithms, especially aimed at improving the treatment of non-localizable epilepsies by targeting the thalamus for neuromodulation. They investigated the thalamograms obtained during SEEG evaluation for epilepsy surgery, using a tool based on temporal Generative Adversarial Networks (TGAN). The authors conclude that this approach can be applied to classify electrographic seizure onset patterns or develop patient-specific seizure detectors from implanted neuromodulation devices.

The section of the Research Topic dedicated to new diagnostic procedures for epilepsies continues with the original study of Irannejad et al. (USA) about mapping of the seizure onset zone (SOZ) with direct cortical stimulation (DCS) in TLE. They show that targeted mapping of SOZ in low amplitude fast activity patterns can better distinguish seizure generators (true responders) from hyperexcitable nodes that may be involved in early propagation.

Finally, this section of the Research Topic ends with the contribution of Suresh et al. (Canada). In their original research they demonstrate a negative association between intraoperative somatosensory evoked potentials amplitude and seizure reduction after VNS implantation. Therefore, they discuss a method for the identification of patients with DRE who are most likely to benefit from VNS.

While most contributions to this Research Topic have focused on surgical treatment or enhanced diagnostic procedures of complex focal epilepsies, three contributions tackle the issue of pharmacological therapy for drug-resistant epilepsies that are not eligible to surgery. Iannone et al. (Italy) describe efficacy and tolerability of add-on treatment with cannabidiol in drug-resistant patients with Dravet syndrome (DS) and Lennox-Gastaut syndrome (LGS). In their systematic review article, Chin et al. (UK) discuss the need for further high-quality international consensus-based treatment guidelines for LGS, DS, and particularly for CDKL5 Deficiency Disorder. In their original research, Li et al. (China) analyze the treatment outcomes of newly diagnosed epilepsy and the risk factors for refractory epilepsy in a population of 466 adult patients. Finally, the last contribution is a perspective article by Ji et al. (China), who performed a meta-analysis on sodium valproate alone or in combination with topiramate (TPM) for treating DRE. They conclude that sodium valproate combined with TPM is more effective than sodium valproate alone.

## Conclusion

In conclusion, this Research Topic embraces various aspects of DRE treatment and provides an up-to-date series of experts' opinions on advanced and new treatment options especially for more complex forms of DRE. Moreover, this article collection offers the possibility to increase the knowledge about unmet needs that might enhance DRE therapy. Finally, it stimulates the identification of new research areas to develop in the field of DRE.

## Author Contributions

GD, VC, EL, RA, and TA edited the Research Topic. All authors contributed and validated the editorial.

## Funding

This work was supported by Italian Ministry of Health Grant NET2013-02355313.

## Conflict of Interest

TA was a consultant and had received consultant fees from NeuroOne, Monteris Medical, and Zimmer Biomet. The remaining authors declare that the research was conducted in the absence of any commercial or financial relationships that could be construed as a potential conflict of interest.

## Publisher's Note

All claims expressed in this article are solely those of the authors and do not necessarily represent those of their affiliated organizations, or those of the publisher, the editors and the reviewers. Any product that may be evaluated in this article, or claim that may be made by its manufacturer, is not guaranteed or endorsed by the publisher.
